# Valyl benzyl ester chloride

**DOI:** 10.1107/S1600536810000176

**Published:** 2010-01-09

**Authors:** Grzegorz Dutkiewicz, B. P. Siddaraju, H. S. Yathirajan, A. N. Mayekar, Maciej Kubicki

**Affiliations:** a Department of Chemistry, Adam Mickiewicz University, Grunwaldzka 6, 60-780 Poznań, Poland; b Department of Chemistry, V. V. Puram College of Science, Bangalore 560 004, India; c Department of Studies in Chemistry, University of Mysore, Manasagangotri, Mysore 570 006, India; d Sequent Scientific limited, New Mangalore 575 011, India

## Abstract

In the title compound (systematic name: 1-benz­yloxy-3-methyl-1-oxobutan-2-aminium chloride), C_12_H_18_NO_2_^+^·Cl^−^, the ester group is approximately planar, with a maximum deviation of 0.040 (2) Å from the least-squares plane, and makes a dihedral angle of 28.92 (16)° with the phenyl ring. The crystal structure is organized by N—H⋯Cl hydrogen bonds which join the two components into a chain along the *b* axis. Pairs of chains arranged anti­parallel are inter­connected by further N—H⋯Cl hydrogen bonds, forming eight-membered rings. Similar packing modes have been observed in a number of amino acid ester halides with a short unit-cell parameter of *ca* 5.5 Å along the direction in which the chains run.

## Related literature

For valsartan, see: Black *et al.* (1997 ▶); Buhlmayer *et al.* (1994 ▶). For related structures, see: Bryndal *et al.* (2006 ▶); Jaeger *et al.* (2003 ▶); Nastopoulos *et al.* (1987 ▶). For a description of the Cambridge Structural Database, see: Allen (2002 ▶). For graph-set motifs, see: Bernstein *et al.* (1995 ▶).
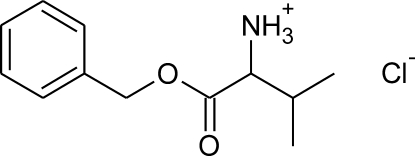


## Experimental

### 

#### Crystal data

C_12_H_18_NO_2_^+^·Cl^−^
*M* *_r_* = 243.72
Monoclinic, 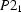

*a* = 9.705 (1) Å
*b* = 5.406 (1) Å
*c* = 13.116 (2) Å
β = 96.58 (1)°
*V* = 683.60 (18) Å^3^
*Z* = 2
Mo *K*α radiation
μ = 0.27 mm^−1^
*T* = 295 K
0.4 × 0.2 × 0.2 mm

#### Data collection

Oxford Diffraction Xcalibur Sapphire2 diffractometer
Absorption correction: multi-scan (*CrysAlis PRO*; Oxford Diffraction, 2009 ▶) *T*_min_ = 0.741, *T*_max_ = 0.948
2649 measured reflections
2010 independent reflections
1652 reflections with *I* > 2σ(*I*)
*R*_int_ = 0.023

#### Refinement

*R*[*F* ^2^ > 2σ(*F* ^2^)] = 0.034
*wR*(*F* ^2^) = 0.077
*S* = 1.06
2010 reflections
159 parameters
1 restraint
H atoms treated by a mixture of independent and constrained refinement
Δρ_max_ = 0.17 e Å^−3^
Δρ_min_ = −0.24 e Å^−3^
Absolute structure: Flack (1983 ▶), 530 Friedel pairs
Flack parameter: 0.02 (8)

### 

Data collection: *CrysAlis PRO* (Oxford Diffraction, 2009 ▶); cell refinement: *CrysAlis PRO*; data reduction: *CrysAlis PRO*; program(s) used to solve structure: *SIR92* (Altomare *et al.*, 1993 ▶); program(s) used to refine structure: *SHELXL97* (Sheldrick, 2008 ▶); molecular graphics: *Stereochemical Workstation Operation Manual* (Siemens, 1989 ▶); software used to prepare material for publication: *SHELXL97*.

## Supplementary Material

Crystal structure: contains datablocks I, global. DOI: 10.1107/S1600536810000176/is2507sup1.cif

Structure factors: contains datablocks I. DOI: 10.1107/S1600536810000176/is2507Isup2.hkl

Additional supplementary materials:  crystallographic information; 3D view; checkCIF report

## Figures and Tables

**Table 1:** Hydrogen-bond geometry (Å, °)

*D*—H⋯*A*	*D*—H	H⋯*A*	*D*⋯*A*	*D*—H⋯*A*
C2—H2⋯O1^i^	0.98	2.38	3.301 (3)	157
N2—H2*A*⋯Cl1^ii^	1.00 (3)	2.26 (4)	3.201 (3)	156 (2)
N2—H2*B*⋯Cl1^iii^	0.90 (3)	2.29 (3)	3.177 (3)	166 (2)
N2—H2*C*⋯Cl1	0.96 (3)	2.15 (3)	3.101 (2)	172.1 (18)
C4—H4*C*⋯Cl1^iv^	0.96	2.95	3.904 (3)	175

Symmetry codes: (i) 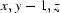; (ii) 
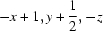
; (iii) 
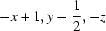
; (iv) 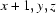.

**Fig. 1.**
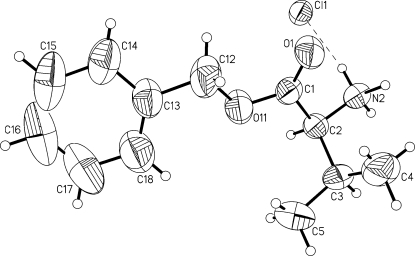
Anisotropic ellipsoid representation of the title compound together with atom labelling scheme. The ellipsoids are drawn at 50% probability level, hydrogen atoms are depicted as spheres with arbitrary radii. Hydrogen bond is shown as dashed line.

**Fig. 2.**
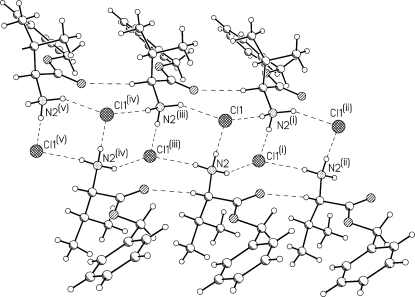
The hydrogen-bonded structure of the title compound. Hydrogen bonds are shown as dashed lines. [Symmetry codes: (i) 1 - x, 1/2 + y, -z; (ii) x, 1 + y, z; (iii) 1 - x, -1/2 + y, -z; (iv) x, -1 + y, z; (v) 1 - x, -3/2 + y, -z.]

## supplementary crystallographic information

### Comment

The title compound (I, Scheme 1), valyl benzyl ester chloride [1-(benzyloxy)-3-methyl-1-oxobutan-2-aminium chloride], is a reactant (Buhlmayer *et al.*, 1994) for the synthesis of valsartan, which belongs to the class of angiotensin II receptor antagonists (Black *et al.*, 1997).

The ester fragment C2/C1/O1/O11/C12 (Fig. 1) is in a good approximation planar, maximum deviation from the least squares plane being 0.040 (2) Å, and it makes a dihedral angle of 28.92 (16)° with the plane of the phenyl ring [planar within 0.009 (3) Å]. The C2—C3 bond is almost perpendicular to the plane of ester group, the torsion angle O11—C1—C2—C3 being -82.2 (3)°.

In the crystal structure, the N—H···Cl hydrogen bonds between the cations and chloride anions join the ionic components into the chains along the *b* direction (Fig. 2 and Table 1). Within these chains there are additional relatively short and linear C—H···O hydrogen bonds involving the C=O oxygen atom. Using graph-set notation (Bernstein *et al.*, 1995), there are two second-order antiparallel C^2^_1_(4) chains which are interconnected by another hydrogen bonds into two different kinds of third-order hydrogen bonded *R*^4^_2_(8) rings. Similar packing was observed in a number of the amino acid ester halides, and it always was connected with the unit-cell parameter of *ca* 5.5 Å. In the Cambridge Structural Database (Allen, 2002), there are 25 organic structures of the amino acid ester halides, and 10 of them display similar crystal packing and appropriate unit-cell parameter. For instance, *L*-tyrosyl methyl ester chloride (Bryndal *et al.*, 2006) crystallizes in *P*2_1_2_1_2_1_ space group with one of the unit-cell parameters 5.424 (2) Å, valyl methyl ester chloride (Jaeger *et al.*, 2003) - also *P*2_1_2_1_2_1_, with 5.894 (2) Å, and (*S*-benzyl-*L*-cysteine methyl ester hydrochloride (Nastopoulos *et al.*, 1987) - in *P*2_1_ with c = 5.211 (2) Å.

The coordination of Cl ion by three hydrogen bonded N—H groups might be described as a trigonal pyramid with N—H groups at the base and Cl ion in the apex. The H···Cl···H angles are in the range 77–118°, and the sum of these angles is 277°. It might be noted that if these coordination is described as tetragonal, the empty coordination place is taken by relatively strong C—H(methyl)···Cl hydrogen bond (Table 1).

### Experimental

The title compound was obtained as a gift sample from Cipla, Bangalore, India. X-ray quality crystals were obtained from slow evaporation of an aqueous solution (m.p. 409–412 K).

### Refinement

Positional and isotropic thermal parameters of the H atoms from the NH_3_ group were freely refined. All other H atoms were put in the calculated idealized positions (C—H = 0.93–0.97 Å) and refined as riding, with *U*_iso_'s set at 1.2 (1.4 for methyl groups) times the *U*_eq_'s of appropriate carrier atoms.

### Crystal data

**Table:**

C_12_H_18_NO_2_^+^·Cl^−^	*F*(000) = 260
*M**_r_* = 243.72	*D*_x_ = 1.184 Mg m^−^^3^
Monoclinic, *P*2_1_	Mo *K*α radiation, λ = 0.71073 Å
Hall symbol: P 2yb	Cell parameters from 1449 reflections
*a* = 9.705 (1) Å	θ = 2.1–26.9°
*b* = 5.406 (1) Å	µ = 0.27 mm^−^^1^
*c* = 13.116 (2) Å	*T* = 295 K
β = 96.58 (1)°	Prism, colourless
*V* = 683.60 (18) Å^3^	0.4 × 0.2 × 0.2 mm
*Z* = 2	

### Data collection

**Table:**

Oxford Diffraction Xcalibur Sapphire2 diffractometer	2010 independent reflections
Radiation source: Nova (Mo) X-ray Source	1652 reflections with *I* > 2σ(*I*)
graphite	*R*_int_ = 0.023
Detector resolution: 5.2679 pixels mm^-1^	θ_max_ = 26.9°, θ_min_ = 2.1°
ω scan	*h* = −10→12
Absorption correction: multi-scan (*CrysAlis PRO*; Oxford Diffraction, 2009)	*k* = −4→6
*T*_min_ = 0.741, *T*_max_ = 0.948	*l* = −15→11
2649 measured reflections	

### Refinement

**Table:**

Refinement on *F*^2^	Secondary atom site location: difference Fourier map
Least-squares matrix: full	Hydrogen site location: inferred from neighbouring sites
*R*[*F*^2^ > 2σ(*F*^2^)] = 0.034	H atoms treated by a mixture of independent and constrained refinement
*wR*(*F*^2^) = 0.077	*w* = 1/[σ^2^(*F*_o_^2^) + (0.040*P*)^2^] where *P* = (*F*_o_^2^ + 2*F*_c_^2^)/3
*S* = 1.06	(Δ/σ)_max_ = 0.001
2010 reflections	Δρ_max_ = 0.17 e Å^−^^3^
159 parameters	Δρ_min_ = −0.24 e Å^−^^3^
1 restraint	Absolute structure: Flack (1983), 530 Friedel pairs
Primary atom site location: structure-invariant direct methods	Flack parameter: 0.02 (8)

### Special details

**Table:**

Geometry. All e.s.d.'s (except the e.s.d. in the dihedral angle between two l.s. planes) are estimated using the full covariance matrix. The cell e.s.d.'s are taken into account individually in the estimation of e.s.d.'s in distances, angles and torsion angles; correlations between e.s.d.'s in cell parameters are only used when they are defined by crystal symmetry. An approximate (isotropic) treatment of cell e.s.d.'s is used for estimating e.s.d.'s involving l.s. planes.
Refinement. Refinement of *F*^2^ against ALL reflections. The weighted *R*-factor *wR* and goodness of fit *S* are based on *F*^2^, conventional *R*-factors *R* are based on *F*, with *F* set to zero for negative *F*^2^. The threshold expression of *F*^2^ > σ(*F*^2^) is used only for calculating *R*-factors(gt) *etc*. and is not relevant to the choice of reflections for refinement. *R*-factors based on *F*^2^ are statistically about twice as large as those based on *F*, and *R*- factors based on ALL data will be even larger.

### Fractional atomic coordinates and isotropic or equivalent isotropic displacement parameters (Å^2^)

**Table:**

	*x*	*y*	*z*	*U*_iso_*/*U*_eq_	
C1	0.6929 (3)	1.0154 (5)	0.2145 (2)	0.0492 (6)	
O11	0.7115 (2)	0.9397 (3)	0.31112 (13)	0.0744 (6)	
C12	0.6987 (5)	1.1297 (7)	0.3890 (2)	0.0963 (12)	
H12A	0.6100	1.2132	0.3756	0.116*	
H12B	0.7719	1.2516	0.3878	0.116*	
C13	0.7099 (4)	1.0030 (7)	0.4919 (2)	0.0766 (10)	
C14	0.6351 (4)	1.0998 (9)	0.5647 (2)	0.1010 (13)	
H14	0.5791	1.2380	0.5506	0.121*	
C15	0.6452 (6)	0.9849 (14)	0.6612 (3)	0.128 (2)	
H15	0.5947	1.0479	0.7114	0.154*	
C16	0.7258 (7)	0.7875 (13)	0.6824 (4)	0.137 (3)	
H16	0.7314	0.7146	0.7469	0.165*	
C17	0.7994 (6)	0.6933 (11)	0.6098 (4)	0.1375 (18)	
H17	0.8544	0.5539	0.6243	0.165*	
C18	0.7931 (5)	0.8035 (8)	0.5143 (3)	0.1053 (14)	
H18	0.8459	0.7411	0.4653	0.126*	
O1	0.6602 (2)	1.2196 (4)	0.18837 (14)	0.0702 (6)	
C2	0.7212 (2)	0.8078 (4)	0.14310 (17)	0.0454 (6)	
H2	0.6851	0.6536	0.1691	0.054*	
N2	0.6429 (2)	0.8642 (6)	0.04138 (15)	0.0476 (5)	
H2A	0.678 (3)	1.021 (7)	0.013 (2)	0.071 (10)*	
H2B	0.649 (3)	0.740 (6)	−0.0041 (19)	0.049 (8)*	
H2C	0.546 (3)	0.867 (7)	0.0479 (16)	0.064 (7)*	
C3	0.8749 (3)	0.7732 (5)	0.1324 (2)	0.0558 (7)	
H3	0.8806	0.6464	0.0796	0.067*	
C4	0.9422 (3)	1.0029 (7)	0.0953 (3)	0.0886 (11)	
H4A	0.9374	1.1335	0.1444	0.124*	
H4B	0.8945	1.0526	0.0304	0.124*	
H4C	1.0375	0.9688	0.0876	0.124*	
C5	0.9560 (3)	0.6756 (7)	0.2299 (3)	0.0844 (10)	
H5A	1.0481	0.6321	0.2163	0.118*	
H5B	0.9103	0.5321	0.2530	0.118*	
H5C	0.9609	0.8010	0.2820	0.118*	
Cl1	0.33538 (6)	0.86692 (12)	0.08481 (4)	0.05096 (19)	

### Atomic displacement parameters (Å^2^)

**Table:**

	*U*^11^	*U*^22^	*U*^33^	*U*^12^	*U*^13^	*U*^23^
C1	0.0545 (16)	0.0364 (15)	0.0597 (15)	−0.0021 (13)	0.0201 (12)	0.0033 (13)
O11	0.1207 (16)	0.0510 (14)	0.0542 (10)	0.0173 (11)	0.0221 (10)	0.0029 (8)
C12	0.171 (4)	0.060 (2)	0.0638 (19)	0.016 (3)	0.040 (2)	−0.0077 (17)
C13	0.107 (3)	0.068 (2)	0.0550 (17)	−0.004 (2)	0.0097 (17)	−0.0008 (16)
C14	0.117 (3)	0.125 (4)	0.062 (2)	−0.008 (3)	0.019 (2)	−0.018 (2)
C15	0.146 (5)	0.179 (6)	0.063 (3)	−0.054 (4)	0.025 (3)	−0.021 (3)
C16	0.181 (6)	0.160 (6)	0.064 (3)	−0.083 (5)	−0.015 (3)	0.021 (3)
C17	0.190 (5)	0.123 (4)	0.089 (3)	0.000 (4)	−0.030 (3)	0.027 (3)
C18	0.143 (4)	0.094 (4)	0.078 (2)	0.013 (3)	0.011 (2)	0.013 (2)
O1	0.1138 (17)	0.0358 (12)	0.0648 (12)	0.0101 (11)	0.0265 (11)	0.0051 (9)
C2	0.0508 (14)	0.0325 (16)	0.0541 (13)	−0.0006 (11)	0.0122 (10)	0.0044 (10)
N2	0.0450 (12)	0.0398 (12)	0.0593 (11)	−0.0043 (17)	0.0117 (9)	−0.0056 (15)
C3	0.0524 (16)	0.0503 (16)	0.0659 (16)	0.0095 (13)	0.0117 (13)	−0.0058 (13)
C4	0.055 (2)	0.090 (3)	0.123 (3)	−0.002 (2)	0.0227 (18)	0.021 (2)
C5	0.066 (2)	0.082 (3)	0.100 (2)	0.0181 (19)	−0.0083 (17)	0.0045 (19)
Cl1	0.0535 (3)	0.0506 (4)	0.0506 (3)	0.0008 (4)	0.0137 (2)	0.0006 (3)

### Geometric parameters (Å, °)

**Table:**

C1—O1	1.188 (3)	C18—H18	0.9300
C1—O11	1.324 (3)	C2—N2	1.489 (3)
C1—C2	1.507 (3)	C2—C3	1.526 (3)
O11—C12	1.464 (4)	C2—H2	0.9800
C12—C13	1.506 (4)	N2—H2A	1.00 (3)
C12—H12A	0.9700	N2—H2B	0.90 (3)
C12—H12B	0.9700	N2—H2C	0.96 (3)
C13—C18	1.359 (5)	C3—C4	1.509 (4)
C13—C14	1.368 (5)	C3—C5	1.518 (4)
C14—C15	1.403 (6)	C3—H3	0.9800
C14—H14	0.9300	C4—H4A	0.9600
C15—C16	1.334 (7)	C4—H4B	0.9600
C15—H15	0.9300	C4—H4C	0.9600
C16—C17	1.353 (8)	C5—H5A	0.9600
C16—H16	0.9300	C5—H5B	0.9600
C17—C18	1.382 (6)	C5—H5C	0.9600
C17—H17	0.9300		
			
O1—C1—O11	124.5 (3)	N2—C2—C3	110.25 (19)
O1—C1—C2	125.0 (2)	C1—C2—C3	113.5 (2)
O11—C1—C2	110.5 (2)	N2—C2—H2	108.6
C1—O11—C12	115.9 (2)	C1—C2—H2	108.6
O11—C12—C13	107.6 (3)	C3—C2—H2	108.6
O11—C12—H12A	110.2	C2—N2—H2A	110.6 (17)
C13—C12—H12A	110.2	C2—N2—H2B	112.0 (16)
O11—C12—H12B	110.2	H2A—N2—H2B	109.2 (19)
C13—C12—H12B	110.2	C2—N2—H2C	109.3 (13)
H12A—C12—H12B	108.5	H2A—N2—H2C	113 (3)
C18—C13—C14	120.1 (4)	H2B—N2—H2C	102 (3)
C18—C13—C12	122.4 (3)	C4—C3—C5	110.8 (3)
C14—C13—C12	117.5 (4)	C4—C3—C2	113.1 (2)
C13—C14—C15	118.2 (5)	C5—C3—C2	112.5 (2)
C13—C14—H14	120.9	C4—C3—H3	106.6
C15—C14—H14	120.9	C5—C3—H3	106.6
C16—C15—C14	121.4 (5)	C2—C3—H3	106.6
C16—C15—H15	119.3	C3—C4—H4A	109.5
C14—C15—H15	119.3	C3—C4—H4B	109.5
C15—C16—C17	119.9 (5)	H4A—C4—H4B	109.5
C15—C16—H16	120.0	C3—C4—H4C	109.5
C17—C16—H16	120.0	H4A—C4—H4C	109.5
C16—C17—C18	120.2 (6)	H4B—C4—H4C	109.5
C16—C17—H17	119.9	C3—C5—H5A	109.5
C18—C17—H17	119.9	C3—C5—H5B	109.5
C13—C18—C17	120.1 (4)	H5A—C5—H5B	109.5
C13—C18—H18	119.9	C3—C5—H5C	109.5
C17—C18—H18	119.9	H5A—C5—H5C	109.5
N2—C2—C1	107.1 (2)	H5B—C5—H5C	109.5
			
O1—C1—O11—C12	−4.1 (4)	C12—C13—C18—C17	−179.8 (4)
C2—C1—O11—C12	175.1 (3)	C16—C17—C18—C13	−1.8 (7)
C1—O11—C12—C13	174.8 (3)	O1—C1—C2—N2	−25.0 (4)
O11—C12—C13—C18	34.5 (5)	O11—C1—C2—N2	155.9 (2)
O11—C12—C13—C14	−147.1 (3)	O1—C1—C2—C3	96.9 (3)
C18—C13—C14—C15	−1.1 (6)	O11—C1—C2—C3	−82.2 (3)
C12—C13—C14—C15	−179.5 (4)	N2—C2—C3—C4	62.6 (3)
C13—C14—C15—C16	0.4 (6)	C1—C2—C3—C4	−57.6 (3)
C14—C15—C16—C17	−0.3 (7)	N2—C2—C3—C5	−170.9 (3)
C15—C16—C17—C18	1.1 (8)	C1—C2—C3—C5	69.0 (3)
C14—C13—C18—C17	1.9 (6)		

### Hydrogen-bond geometry (Å, °)

**Table:**

*D*—H···*A*	*D*—H	H···*A*	*D*···*A*	*D*—H···*A*
C2—H2···O1^i^	0.98	2.38	3.301 (3)	157
N2—H2A···Cl1^ii^	1.00 (3)	2.26 (4)	3.201 (3)	156 (2)
N2—H2B···Cl1^iii^	0.90 (3)	2.29 (3)	3.177 (3)	166 (2)
N2—H2C···Cl1	0.96 (3)	2.15 (3)	3.101 (2)	172.1 (18)
C4—H4C···Cl1^iv^	0.96	2.95	3.904 (3)	175

Symmetry codes: (i) *x*, *y*−1, *z*; (ii) −*x*+1, *y*+1/2, −*z*; (iii) −*x*+1, *y*−1/2, −*z*; (iv) *x*+1, *y*, *z*.
